# Daratumumab-Based Therapeutic Approaches and Clinical Outcomes in Multiple Myeloma and other Plasma Cell Dyscrasias: Insights from a Nationwide Real-World Chart Review Study

**DOI:** 10.46989/001c.124362

**Published:** 2024-10-11

**Authors:** Allison C. Y. Tso, Wee Joo Chng, Yeow Tee Goh, Melissa G Ooi, Yunxin Chen, Chandramouli Nagarajan, Daryl Tan, Sanchalika Acharyya, Kiat Hoe Ong

**Affiliations:** 1 Haematology Tan Tock Seng Hospital; 2 Haematology National University Cancer Institute, Singapore; 3 Haematology Singapore General Hospital; 4 Clinic for Lymphoma, Myeloma and Blood Disorders; 5 Clinical Research & Innovation Office Tan Tock Seng Hospital

**Keywords:** daratumumab, real-world, multiple myeloma, Asian, plasma cell dyscrasia

## Abstract

Singapore leads Southeast Asia in the routine use of daratumumab for multiple myeloma and other plasma cell dyscrasias. This retrospective review analyzed 112 patients who received daratumumab between 2012 and 2020. Tolerability, and efficacy based on prior lines (PL) of therapy, cytogenetic risk group, and the presence of renal impairment were presented. Infusion-related reactions occurred in 26.8% of patients. Grades 1 and 2 hematological and non-hematological adverse events were observed in 14.3% and 33.9% of patients, respectively. After a median follow-up of 16.9 months, there was no significant difference in overall response rates (ORR) (86% versus 76.3%, p = 0.082) or depth of response (≥ complete response (CR), 35.1% versus 28.9%, p = 0.469) between myeloma patients with and without renal dysfunction. Newly diagnosed and relapsed/refractory patients had an ORR of 92% and 76.3%, and a ≥ VGPR (very good partial response) rate of 80% and 55.3%, respectively. Median progression-free survival (PFS) was better for patients with 0/1 PL compared to ≥ 2 PLs (19.8 versus 6.2 months, p < 0.001), with a deeper response (≥ CR, 38.5% versus 16.7%, p = 0.033). Forty-six and a half percentage of patients had high-risk FISH abnormalities, and those with 0/1 PL had a significantly better ORR than those with ≥ 2 PLs (83.3% vsersus 47.1%, p = 0.022), achieving an ORR similar to that of the general cohort (80.2%, p = 0.905). In conclusion, positioning daratumumab in earlier lines of therapy leads to better outcomes and may mitigate the impact of high-risk FISH abnormalities.

## Background

Multiple myeloma accounts for about 2% of all malignancies and 10% of hematological cancers, primarily affecting the elderly, with 43% of new cases in the UK occurring in patients aged 75 and over.^1^ In Singapore, the incidence is around 1.69 per 100,000 people,^2^ while the rate in Southeast Asia is 0.96 per 100,000.^3^ Treatment for myeloma has evolved substantially over the decades. In the 1960s, treatment centered on oral melphalan and prednisolone. The 1980s introduced VAD (vincristine, doxorubicin, dexamethasone) chemotherapy^4^ and high-dose dexamethasone. The 1990s established thalidomide and autologous stem cell transplantation as standards of care. In 2003, the first proteosome inhibitor (PI), bortezomib, became available, followed by the immunomodulatory drug (IMID) lenalidomide in 2006. The 2010s saw the introduction of second-generation PIs carfilzomib and ixazomib, and the third-generation IMID pomalidomide. Between 1990 and 2004, survival rates improved significantly,^5^ although patients refractory to both a PI and an IMID have a median overall survival (OS) of 8.5 months.^6^

The anti-CD38 monoclonal antibody daratumumab, which works through immune-mediated mechanism,^7–9^ has been approved both as a monotherapy^10^ and in combination with other therapies^11–21^ for multiple myeloma, demonstrating significant improvements in progression-free (PFS) and overall survival (OS).^22–24^ There has been an increasing trend in the upfront use of daratumumab-based quadruplets^25–29^ to achieve minimal residual disease (MRD) negative status, which is considered a surrogate marker for improved OS^30^ and PFS.^30–32^ Daratumumab is also used in light chain amyloidosis (AL)^33^ and other plasma cell disorders.^34–38^ Although around 45 - 48% of patients experience infusion-related reactions, serious adverse events are uncommon.^11,12,39,40^ Daratumumab can affect stem cell harvest, often requiring the use of plerixafor (CXCR4 antagonist) for mobilization,^41,42^ which has cost implications.^43^

Within Southeast Asia, Singapore is at the forefront of adopting daratumumab, following its regulatory approval for myeloma in October 2016 and for AL amyloidosis in February 2022, supported by guidelines published by the Singapore Myeloma Study Group.^2,44^ The spiralling cost of cancer drugs underscores the importance of real-world analyses of local healthcare costs and health technology assessments to inform stakeholders.^45^ In 2022, the Ministry of Health of Singapore introduced the Cancer Drug List (CDL), a compilation of cost-effective and clinically proven cancer drugs and regimens eligible for varying levels of government subsidies and insurance coverage. The CDL provides clinical guidance, standardization, and accessibility, ensuring the long-term financial sustainability of Singapore’s healthcare system.^46^ At the time of writing, daratumumab is not listed in the CDL for combination therapy with carfilzomib, pomalidomide, or the combination of bortezomib and lenalidomide, as it is not considered as cost-effective as other available standard drugs and combinations. Although these regimens can be prescribed, their costs typically need to be borne by the patient, influencing on physicians’ prescribing patterns towards more cost-effective treatments. Paired with efficacy data, if the cost of daratumumab decreases or biosimilars become available, it may be considered for future CDL listings.

This paper aims to share the approach of how Singaporean physicians incorporate daratumumab into the treatment algorithm for multiple myeloma, prior to the launch of the CDL. We observed the tolerability of daratumumab-based therapies, including rates of infusion-related reactions, adverse event, and the impact on subsequent stem cell collection. We report on treatment efficacy and outcomes based on the number of prior therapy lines, cytogenetic risk group, and the presence or absence of renal impairment. For patients who subsequently relapsed, we examined the indicators of relapse, the presence of extramedullary disease, their subsequent therapies, and PFS 2 (PFS2). Our goal is to share real-world experience of daratumumab among Asian subjects, including those with severe renal impairment, to complement data from randomized controlled trials.

## Method

### a. Study Design and Data Source

This retrospective study includes data from all three university teaching hospitals in Singapore, focusing on adult patients age 21 and above (the age of majority in Singapore) who received daratumumab between February 2012 and December 2020. Patients participating in clinical trials were excluded. Some patients received daratumumab prior to FDA approval through early access and compassionate programs. Approval was obtained from the National Healthcare Group (NHG) Institutional Review Board (2020/00814). De-identified patient data were sourced from each institution’s electronic medical records, with a data cut-off date of 30^th^ September 2021.

### b. Endpoint Definitions and Assessments

Functional status was evaluated using the Eastern Cooperative Oncology Group (ECOG) performance status scale. Renal impairment was defined as creatine clearance (CrCl) < 60 mL/min. Treatment response and disease relapse were determined using the International Myeloma Working Group (IMWG) uniform response criteria. Overall response rate (ORR) was defined as achieving a partial response (PR) or better (≥ PR). PFS was the time from daratumumab initiation to disease progression or death, with censoring of patients lost to follow-up. OS was from the start of daratumumab treatment to death or the last follow-up date. ORR, PFS and OS were the primary efficacy endpoints. Adverse events (AEs) were graded by Common Terminology Criteria for Adverse Events (CTCAE) version 5.0.

### c. Clinical Data Collection

Data collected included patient demographics, Eastern Cooperative Oncology Group (ECOG) status, diagnoses, and disease characteristics. Myeloma patients were staged according to the IMWG International Staging System (ISS) and Revised ISS (R-ISS). Identified high-risk fluorescence in situ hybridization (FISH) abnormalities included t(4;14), del(17/17p), t(14;16), t(14;20), loss of TP53, and chromosome 1 abnormalities (gain/amp(1q21), del(1p)). Laboratory parameters before daratumumab initiation included full blood count, creatinine levels, calculated CrCl using Cockcroft-Gault equation, and corrected calcium levels. We documented prior therapies received, treatment refractoriness, stem cell transplantation history, and concurrent myeloma therapies. The best response post-daratumumab, including minimal residual disease (MRD) by next-generation flow cytometry (NGF) with a sensitivity of 1 in 10^5^ nucleated cells, was documented. Response rates were compared based on prior therapy lines, FISH abnormalities and the presence or absence of renal failure. All infusion-related reactions (IRRs), and hematological and non-hematological AEs from daratumumab initiation to the cut-off date were recorded. For subsequent stem cell transplantation, stem cell quantity and plerixafor use were noted. In patients with hepatitis B exposure, the use of hepatitis B prophylaxis was recorded. We recorded the characteristics and outcomes of patients who subsequently relapsed, including indicators of relapse, presence of extramedullary disease, subsequent therapies, and PFS 2.

### d. Statistical Analysis

Demographic characteristics, clinical presentations, treatment courses, and outcomes were summarized using appropriate statistical measures. Survival probabilities were estimated using the Kaplan–Meier method, and PFS and OS distributions compared using the log-rank test. Categorical outcomes, such as response rates, were compared using the chi-squared test. A p-value of < 0.05 was considered significant. All analyses were performed in R version 4.3.3, using the “survival” package for survival analysis.

## Results

### a) Patient characteristics

From February 2012 to December 2020, a total of 112 patients received daratumumab. Among them, 51 were from Tan Tock Seng Hospital, 27 from National University Hospital, and 34 from Singapore General Hospital. Ethnically, the cohort consisted of 66.1% Chinese, 16.9% Malay, and 11.6% Indian patients. Most patients had a functional status of ECOG 0 (60.7%) or ECOG 1 (25.9%). A total of 101 (90.2%) had multiple myeloma, six had AL amyloidosis, three had plasma cell leukemia, one had POEMS (polyneuropathy, organomegaly, endocrinopathy, monoclonal plasma cell disorder, skin changes) syndrome, and one had PGNMID (proliferative glomerulonephritis with monoclonal immunoglobulin deposits). Twenty patients tested positive for hepatitis B core total antibody; one had detectable hepatitis B surface antigen. All 20 tested negative for hepatitis B virus (HBV) DNA. Fifteen of these 20 patients received prophylactic entecavir for a median duration of 638 days (range 14 – 2316 days), with no reported cases of hepatitis B reactivation. Table 1 summarizes the characteristics of the 101 patients diagnosed with multiple myeloma including 57 males and 44 females with a median age of 64 years. Renal impairment was present in 56.4% of patients, with 26.7% having a CrCl of < 30 mL/mins. Additionally, 39 patients (38.6%) were categorized as R-ISS stage III, and 47 (46.5%) had high-risk FISH abnormalities. The most prevalent FISH abnormality was aberrations of chromosome 1, present in all patients with double and triple-hit myeloma. Among the 35 patients with aberrations of chromosome 1, 60% were categorized as R-ISS stage III.

**Table 1.** Demographics and disease characteristics of patients with multiple myeloma.

**Characteristic**	**n = 101**	**Characteristic**	**n = 101**
Median age (range) – year	64 (33-89)	R-ISS disease stage – n (%)	
<65 year - n (%)	62 (61.4)	I	12 (11.9)
65-74 year - n (%)	32 (31.7)	II	42 (41.6)
≥75 year - n (%)	7 (6.9)	III	39 (38.6)
Gender: male - n (%)	57 (56.4)	Not known	8 (7.9)
female - n (%)	44 (43.6)	Cytogenetic profile – n (%)	
Renal impairment - n (%),	59 (58.4)	High-risk FISH	47 (46.5)
CrCl ≥ 60 mL/min	38 (37.6)	- Single hit	28 (59.6)
CrCl 30-59 mL/min	20 (19.8)	- Double hit	17 (36.2)
CrCl <30 mL/min	27 (26.7)	- Tiple hit	2 (4.3)
CrCl <15 mL/min	9 (8.9)	Non-high-risk FISH	49 (48.5)
Unknown CrCl	6 (5.9)	Not known	5 (5)
Median CrCl mL/min, (range)	35 (5 – 147)	Median time since diagnosis (months, range)	14 (0 – 138)
Type of disease - n (%)		Median lines of prior therapy (range)	1 (0 – 4)
IgG	57 (56.4)	Newly diagnosed myeloma – n (%)	25 (24.8)
IgA	21 (20.8)	Relapsed/ refractory disease – n (%)	76 (75.2)
IgD	3 (3)	Previous therapy – n (%)	n = 76
Light chain only	18 (17.8)	PI only	74 (97.4)
Non-secretory	1 (1)	IMIDs only	55 (72.4)
Unknown	1 (1)	Corticosteroids	76 (100)
ISS disease stage – n (%)		Alkylating agents	53 (69.7)
I	17 (16.8)	Stem cell transplantation	33 (43.4)
II	31 (30.7)	Refractory disease – n (%)	n = 76
III	48 (47.5)	To last line of therapy	45 (59.2)
Not known	5 (5)	To PI only	18 (23.7)
		To IMIDs only	14 (18.4)
		To PI and IMIDs	15 (19.7)

### b) Subgroup Analysis of Myeloma Patients

#### i) Treatment prior to daratumumab

Of the 101 myeloma patients, 25 (24.8%) were newly diagnosed and had not received prior treatment. Among the 76 relapsed/refractory patients, 74 (97.4%) had prior exposure to PIs, and 55 (72.4%) had been treated with IMIDs. A total of 23.7% of patients were refractory to PIs alone, 18.4% to IMIDs alone, and 19.7% to both PIs and IMIDs. Additionally, 43.4% of patients had undergone prior autologous stem cell transplantation. The median number of prior lines (PL) of therapy was 1.

#### ii) Daratumumab-containing regimens

The time from diagnosis to the initiation of daratumumab ranged from 0 to 138.5 months (mean: 23 months, median: 14.3 months). Of the 101 myeloma patients, 89 (88.1%) received combination therapy, including 24 of the 25 newly diagnosed patients. The immunochemotherapy regimens and their abbreviations can be found in Table 2, and the most prevalent regimens prescribed were D-Rd (n = 38, 37.6%), D-Vd (n = 23, 22.8%), and D-Pd (n = 6, 5.9%).

**Table 2.** Chemotherapy regimens used in newly diagnosed and relapsed/refractory multiple myeloma patients.

**Regimen Abbreviation**	**Regimen combinations**	**Newly diagnosed patients, n = 25 (%)**	**Relapsed/ refractory patients, n = 76 (%)**	**Total myeloma patients n = 101 (%)**
D-Rd	daratumumab, lenalidomide, dexamethasone	8 (32%)	30 (39.5%)	38 (37.6%)
D-Vd	daratumumab, bortezomib, dexamethasone	4 (16%)	19 (25%)	23 (22.8%)
D-Pd	daratumumab, pomalidomide, dexamethasone	0	6 (7.9%)	6 (5.9%)
D-Kd	daratumumab, carfilzomib, dexamethasone	0	5 (6.6%)	5 (5%)
D-VTd	daratumumab, bortezomib, thalidomide, dexamethasone	5 (20%)	0	5 (5%)
D-VRd	daratumumab, bortezomib, lenalidomide, dexamethasone	3 (12%)	1 (1.3%)	4 (4%)
D-VCd	daratumumab, bortezomib, cyclophosphamide, dexamethasone	2 (8%)	1 (1.3%)	3 (3%)
D-Id	daratumumab, ixazomib, dexamethasone	0	1 (1.3%)	1 (1%)
D-VMP	daratumumab, bortezomib, melphalan, prednisolone	1 (4%)	0	1 (1%)
D-VRD-PACE	daratumumab, bortezomib, lenalidomide, dexamethasone, cisplatin, doxorubicin, cyclophosphamide, and etoposide	1 (4%)	0	1 (1%)
D-Td	daratumumab, thalidomide, dexamethasone	0	2 (2.6%)	2 (2%)
D	Daratumumab monotherapy	1 (4%)	11 (14.5%)	12 (11.9%)

#### iii) Efficacy of daratumumab-based therapy

**Figure 1.**
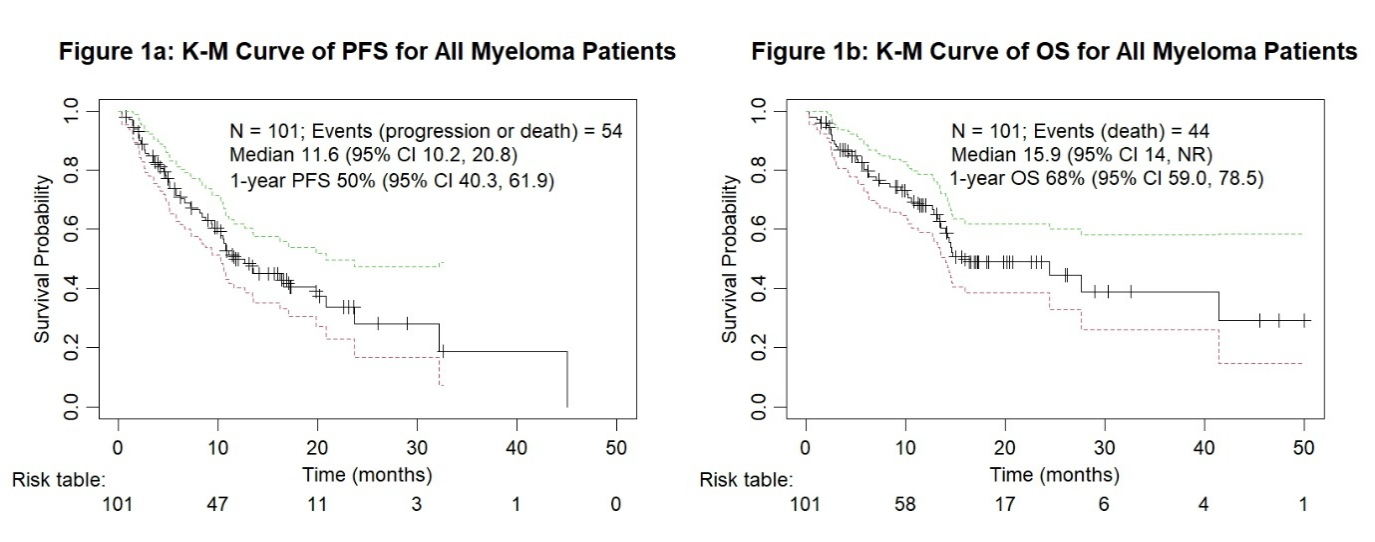
Kaplan-Meier survival analysis for all myeloma patients

Figures 1a and 1b illustrate the PFS and OS of 101 myeloma patients treated with daratumumab-based therapy. After a median follow-up of 16.9 months, 56 patients (55.4%) were alive, and 44 (43.6%) had died. The 12-month PFS rate was 50% (95% CI 40.3, 61.9), with a median of 11.6 months (95% CI 10.2, 20.8). Median OS was 15.9 months (95% CI 14.0, not reached), with a 12-month OS rate of 68% (95% CI 59.0, 78.5). The ORR was 80.2%, and 61.4% patients achieved very good partial response or better (≥ VGPR).

**Figure 2.**
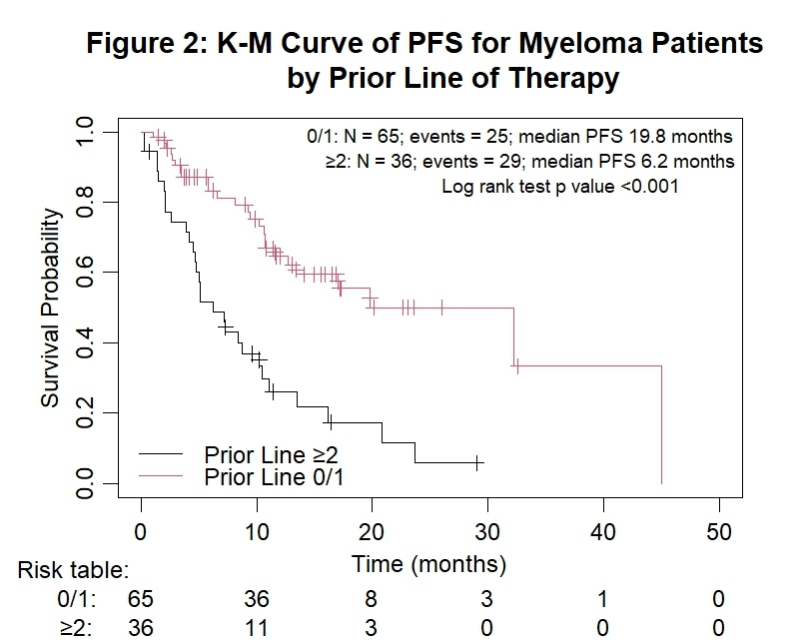
Kaplan-Meier survival analysis for all myeloma patients by subgroups of number of prior lines of therapy

Response rates categorized by PL of therapy are presented in Table 3. Among the 25 newly diagnosed myeloma patients, the ORR was 92%, the rate of complete response or better (≥ CR) was 36%, ≥ VGPR rate was 80%, and both patients with stringent CR (sCR) achieved MRD negativity by flow cytometry. The depth of response among the 76 relapsed/refractory myeloma patients decreased with each additional line of therapy, with an ORR of 76.3%, ≥ CR rate of 28.9%, and ≥ VGPR rate of 55.3%. The outcomes of the 65 patients who received daratumumab-based treatment either upfront or at first relapse (0/1 PL), were compared to those of the 36 who received it at later lines of therapy (≥ 2 PLs). They achieved superior and deeper responses with an ORR 90.7% versus 61.1% (p < 0.001), ≥ CR of 38.5% versus 16.7% (p = 0.033), and ≥ VGPR 69.2% versus 47.2% (p = 0.031). The median PFS was significantly longer at 19.8 months (95% CI 13.4, not reached) compared to 6.2 months (95% CI 4.7, 11.1); p < 0.001 (Figure 2). Among the 81 responders (≥ PR), the median PFS was 32.2 months versus 10.5 months for patients with 0/1 PL and ≥ 2 PLs, respectively (p = 0.003). Among the 62 patients who attained ≥ VGPR, the median PFS was 32.2 months versus 11.1 months for patients with 0/1 PL and ≥ 2 PLs, respectively (p = 0.008).

**Table 3.** Response to daratumumab among 101 myeloma patients according to the number of prior lines (PL) of therapy.

	**0 PL**	**1 PL**	**2 PL**	**3 PL**	**4 PL**	**1-4 PL**	**0/1 PL**	**≥ 2 PL**
Total patients, n	25	40	22	12	2	76	65	36
Overall response ORR, n (%)	23 (92)	36 (90)	16 (72.7)	5 (41.7)	1 (50)	58 (76.3)	59 (90.7)	22 (61.1)
CR or better, n (%)	9 (36)	16 (40)	5 (22.7)	0	1 (50)	22 (28.9)	25 (38.5)	6 (16.7)
Stringent CR, n (%)	2 (8)	2 (5)	0	0	0	2 (2.6)	4 (6.2)	0
CR, n (%)	7 (28)	14 (35)	5 (22.7)	0	1 (50)	20 (26.3)	21 (32.3)	6 (16.7)
VGPR or better, n (%)	20 (80)	25 (62.5)	12 (54.5)	4 (33.3)	1 (50)	42 (55.3)	45 (69.2)	17 (47.2)
VGPR, n (%)	11 (44)	9 (22.5)	7 (31.8)	4 (33.3)	0	20 (26.3)	20 (30.8)	11 (30.6)
PR, n (%)	3 (12)	11 (27.5)	4 (18.2)	1 (8.3)	0	16 (21.1)	14 (21.5)	5 (13.9)
MR, n (%)	0	1 (2.5)	3 (13.6)	0	1 (50)	5 (6.6)	1 (1.5)	4 (11.1)
SD, n (%)	0	0	1 (4.5)	2 (16.7)	0	3 (3.9)	0	3 (8.3)
PD, n (%)	1 (4)	2 (5)	2 (9.1)	5 (41.7)	0	9 (11.8)	3 (4.6)	7 (19.4)
Unknown, n (%)	1 (4)	1 (2.5)	0	0	0	1 (1.3)	2 (3.1)	0

**Figure 3.**
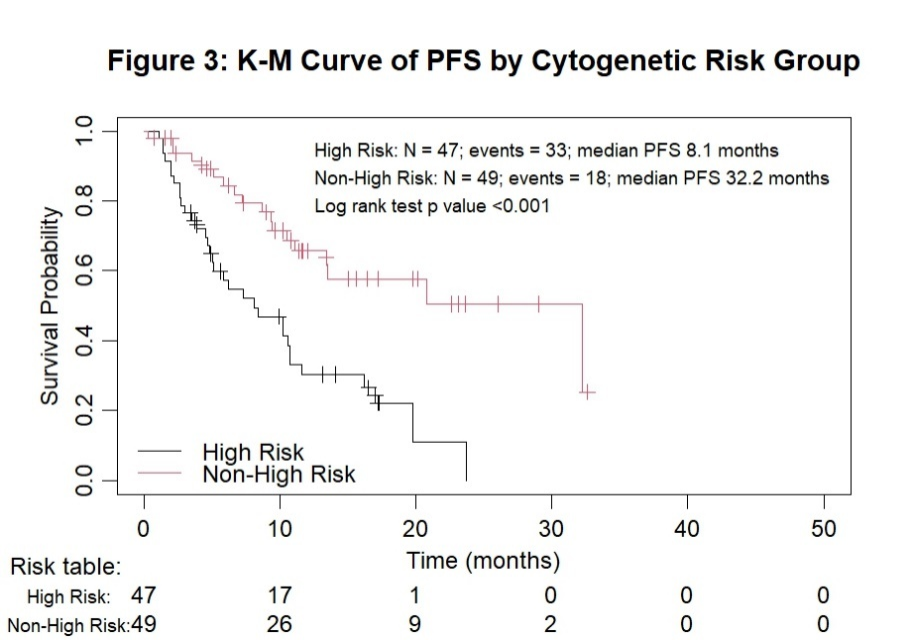
Kaplan-Meier survival analysis for all myeloma patients by subgroups of cytogenetic risk

Table 4 illustrates the superior response rates of non-high-risk patients compared to high-risk ones in both newly diagnosed and relapsed/refractory myeloma settings. In the cohort, the median PFS was 8.1 (95% CI 5.0, 11.6) and 32.2 months (95% CI 13.4, not reached) for the high-risk and non-high-risk groups, respectively (p < 0.001; Figure 3). Among the 25 newly diagnosed myeloma patients, the ORR was 100% versus 83.3% for non-high-risk and high-risk patients, respectively. Among the 71 evaluable relapsed/refractory myeloma patients, the ORR was 86.1% versus 65.7% for non-high-risk and high-risk patients, respectively.

**Table 4.** Response to daratumumab among the 25 newly diagnosed myeloma patients (NDMM) and the 71 evaluable relapsed/refractory myeloma patients (RRMM) according to cytogenetic risk stratification and prior lines (PL) of therapy.

	**NDMM HR**	**NDMM non-HR**	**RRMM HR**	**RRMM non-HR**	**High risk (All)**	**Non-high risk (All)**	**High risk 0/1 PL**	**Non high risk 0/1 PL**	**High risk ≥ 2 PL**	**Non high risk ≥ 2 PL**
Total patients, n	12	13	35	36	47	49	30	31	17	18
Overall response ORR, n (%)	10 (83.3)	13 (100)	23 (65.7)	31 (86.1)	33 (70.2)	44 (89.8)	25 (83.3)	30 (96.8)	8 (47.1)	14 (77.8)
CR or better, n (%)	7 (58.3)	2 (15.4)	10 (28.6)	12 (33.3)	17 (36.2)	14 (28.6)	14 (46.7)	11 (35.4)	3 (17.6)	2 (11.1)
Stringent CR, n (%)	1 (8.3)	1 (7.7)	2 (5.7)	0	3 (6.4)	1 (2)	3 (10)	1 (3.2)	0	0
CR, n (%)	6 (50)	1 (7.7)	8 (22.9)	12 (33.3)	14 (29.8)	13 (26.5)	11 (36.7)	10 (32.3)	3 (17.6)	3 (16.7)
VGPR or better, n (%)	10 (83.3)	10 (76.9)	16 (45.7)	24 (66.7)	26 (55.3)	34 (69.4)	20 (66.7)	23 (74.2)	6 (35.3)	10 (55.6)
VGPR, n (%)	3 (25)	8 (61.5)	6 (17.1)	12 (33.3)	9 (19.1)	20 (40.8)	6 (20)	12 (38.7)	3 (17.6)	8 (44.4)
PR, n (%)	0	3 (23.1)	7 (20)	7 (19.4)	7 (14.9)	10 (20.4)	5 (16.7)	7 (22.6)	2 (11.8)	3 (16.7)
MR, n (%)	0	0	3 (8.6)	2 (5.6)	3 (6.4)	2 (4.1)	0	1 (3.2)	3 (17.6)	1 (5.6)
SD, n (%)	0	0	0	2 (5.6)	0	2 (4.1)	0	0	0	2 (11.1)
PD, n (%)	1 (8.3)	0	8 (22.9)	1 (2.8)	9 (19.1)	1 (2)	3 (10)	0	6 (35.3)	1 (5.6)
Unknown, n (%)	1 (8.3)	0	1 (2.9)	0	2 (4.3)	0	2 (6.7)	0	0	0

**Figure 4.**
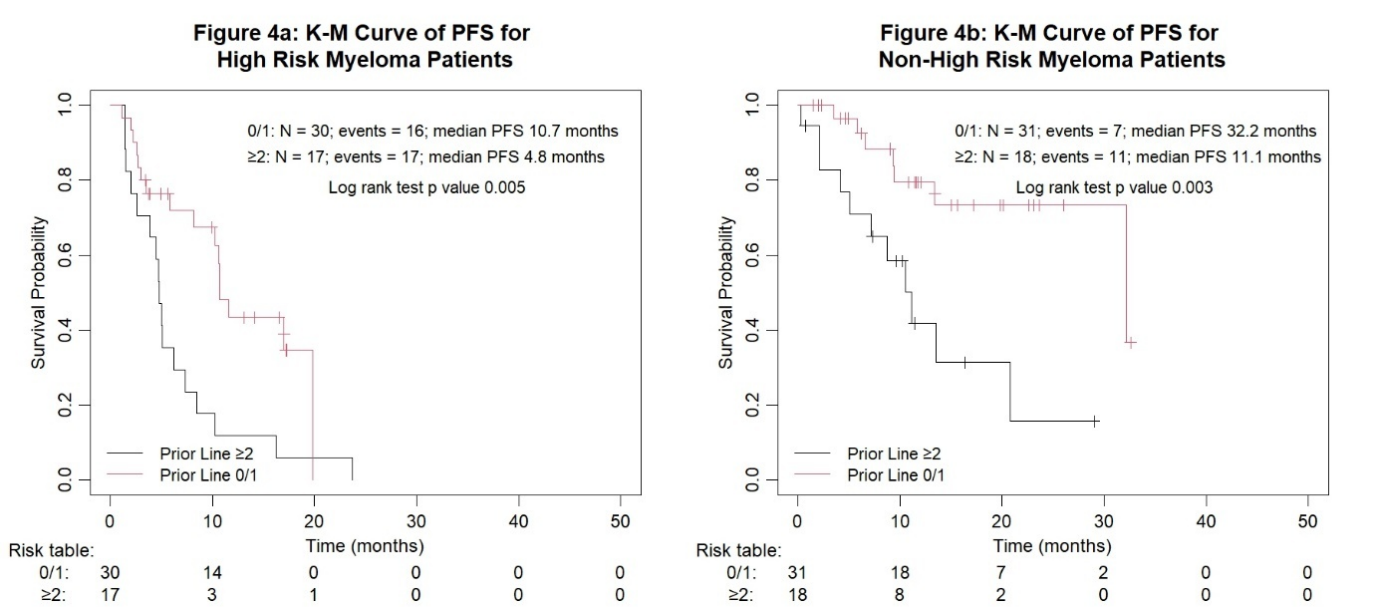
Kaplan-Meier survival analysis for all myeloma patients by subgroups of cytogenetic risk and number of prior lines of therapy

The PFS curves by cytogenetic risk and number of prior lines of therapy are presented in Figures 4a and 4b.

**Figure 5.**
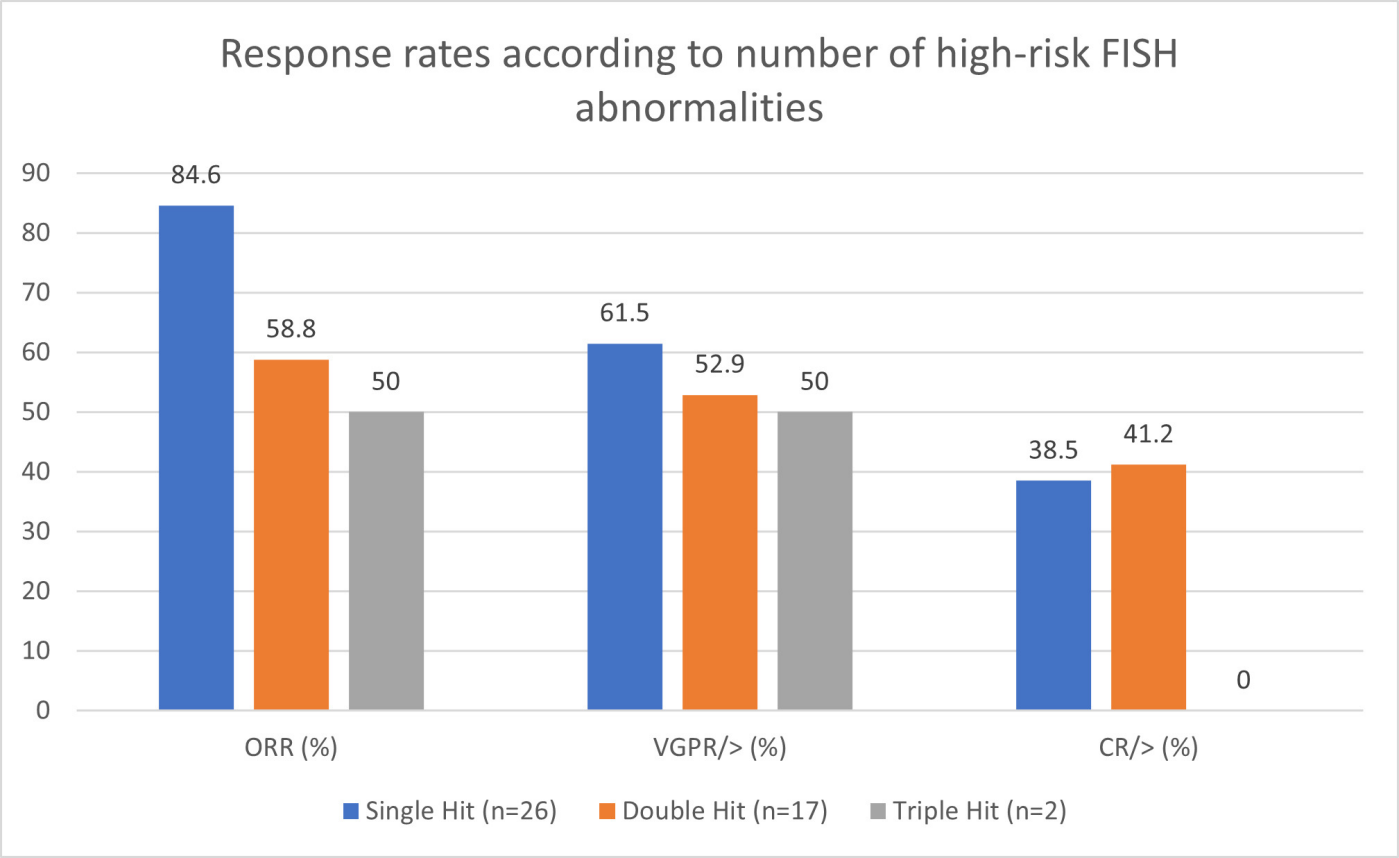
Response rates according to the number of high-risk FISH abnormalities

Among the 47 patients with high-risk disease, 30 received 0/1 PL, with ORR of 83.3%, ≥ CR 46.7%, and ≥ VGPR 66.7%. When compared with the 17 high-risk patients who received ≥ 2 PL, their ORR dropped to 47.1% (p = 0.022), with ≥ CR at 17.6% (p = 0.050), and ≥ VGPR at 35.3% (p = 0.031). The ORR of high-risk patients with one FISH abnormality (single-hit), two abnormalities (double-hit) and three abnormalities (triple-hit) were 84.6%, 58.8% and 50%, respectively (Figure 5). Among the 49 non-high-risk patients, those with 0/1 PL fared better than those with ≥ 2 PL with an ORR of 96.8%, ≥ CR of 35.5%, and ≥ VGPR of 74.2%; compared to an ORR of 77.8% (p = 0.103), ≥ CR 16.7% (p = 0.281), and ≥ VGPR 61.1% (p = 0.524).

Eighteen of the 101 myeloma patients (17.8%) subsequently underwent autologous stem cell transplantation after daratumumab-based therapy. This included 55.6% of all newly diagnosed patients, 38.9% with 1 PL, and 5.6% with 2 PLs. At the time of analysis, 53 patients (52.5%) remained in remission, while 37 (36.6%) experienced relapse, and 10 (9.9%) had progression of disease (PD) on daratumumab. Among the 37 patients who experienced relapse, 2 were newly diagnosed. Fourteen patients (37.8%) collectively experienced 23 events of extramedullary relapses, including plasmacytomas affecting the nervous system, retroperitoneum, ureter, pleura, bones, muscles, and subcutaneous tissues. One patient had plasma cells in the cerebrospinal fluid, 3 had plasma cell leukemia, and 3 had myelomatous pleural effusion. Discordance between biochemistry and/or pathology results and clinical signs of relapse was observed in 5 of the 37 patients. In three cases, serum free light chains were either normal or improving, despite patients developing clinical relapse with plasmacytomas or plasma cell leukemias. In 2 other patients, no increase in plasma cells were seen in the bone marrow biopsies, but one had biopsy-proven plasmacytomas of the scalp, and one had new cytogenetic evolution with the emergence of multiple plasmacytomas.

We identified a subset of 10 ultra high-risk patients who experienced PD on daratumumab-based therapy, half of whom belonged to R-ISS stage III, 90% had high-risk FISH abnormalities, and 60% had either double or triple-hit myeloma. Furthermore, 80% of these patients harboured chromosome 1 abnormalities, while 40% had loss of TP53 by FISH analysis. As of the data cut-off date, 9 out of the 10 patients were deceased with a median OS of only 4.1 months.

#### iv) Subsequent lines of treatment after failing daratumumab

Among the 47 patients who relapsed or progressed on daratumumab, 33 (70.2%) pursued further treatment, while 12 (25.5%) died from disease progression, and 2 (4.3%) from sepsis. As of the data cut-off, 11 patients (23.4%) were still alive, and 36 (76.6%) had passed away. Among those who pursued further treatment, 4 participated in clinical trials with venetoclax and pomalidomide-based therapy. Six received carfilzomib-based therapy, 4 underwent E-Pd (elotuzumab, pomalidomide, and dexamethasone), and 3 each received D-Rd and K-Pd (carfilzomib, pomalidomide and dexamethasone). Two patients each received D-Pd, V-Pd (bortezomib, pomalidomide and dexamethasone), and VPom-DCEP (bortezomib, pomalidomide, dexamethasone, cyclophosphamide, etoposide, cisplatin). One patient each received selinexor, bendamustine, doxorubicin, venetoclax, lenalidomide and VD-PACE (bortezomib, dexamethasone, cisplatin, doxorubicin, cyclophosphamide, etoposide). Among these 33 patients, 16 (48.5%) subsequently relapsed. Given the short follow-up period, the median PFS2 was only 3 months (mean 5, range 0.3 – 23.2).

#### v) Renal Impairment

**Figure 6.**
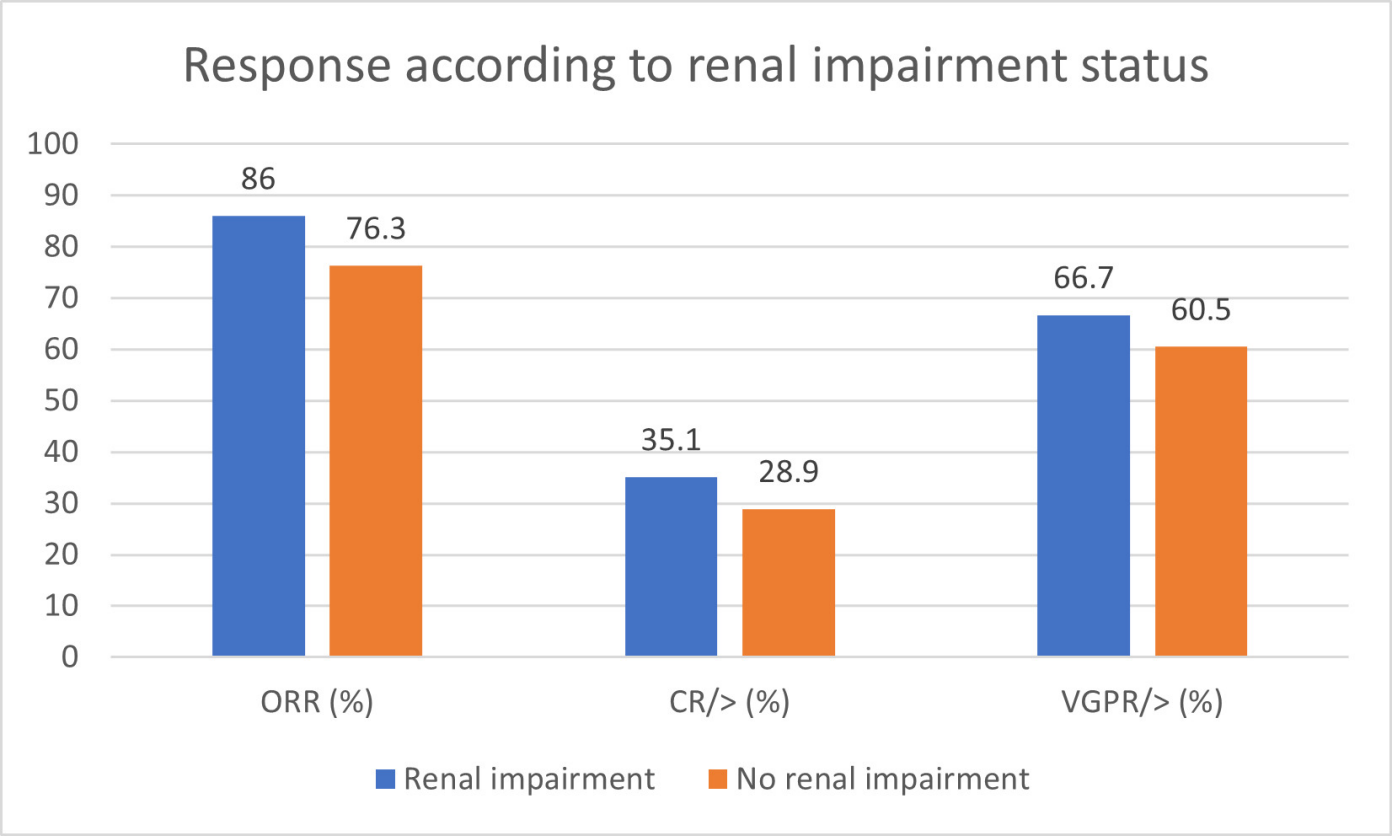
Response according to renal impairment status

The response rates for 56 of the 57 patients with renal impairment are illustrated in Figure 6. Their ORR was 86%, with 35.1% achieving ≥ CR, and 66.7% achieving ≥ VGPR, compared to an ORR of 76.3%, with 28.9% achieving ≥ CR, and 60.5% achieving ≥ VGPR, in patients with normal renal function. Although the sample size was small, our findings suggest that renal status does not significantly impact response rates to daratumumab-based treatment (p = 0.387).

### d) Safety and Efficacy

Infusion-related reactions (IRRs) occurred in 26.8% of patients, mainly during the first dose, and were mostly grades 1 and 2 (Table 5). No IRRs occurred beyond the second infusion. The most frequent IRRs were pyrexia and chills/rigors, followed by dyspnoea and hypotension. Daratumumab was discontinued in only one patient who experienced grade 4 laryngeal edema and hypotension. Hematological AEs were reported in 16 patients (14.3%). All three patients with grade 3 or 4 neutropenia received daratumumab in combination with an IMID. Four patients required temporary treatment cessation and all subsequently resumed treatment without further events. Non-hematological AEs were observed in 38 patients (33.9%), with infections, peripheral sensory neuropathy, pyrexia, and dyspnoea being the most common. Eighteen patients required temporary therapy interruption. Only one patient, who experienced grade 4 pneumonia, required permanent cessation of daratumumab.

**Table 5.** Infusion related reactions (IRRs) and treatment emergent adverse events (AEs) among 112 patients who received daratumumab.

**Infusion-related reactions (IRRs)**	**N (%)**	**Non-Hematological adverse events (AEs)**	**N (%)**
No IRRs	78 (69.6)	No non-hematological AEs	72 (64.3)
Unknown	3 (2.7)	Unknown	2 (1.8)
IRRs - n (%)	30 (26.8)	Non-Hematological AEs (unique patients)	38 (33.9)
- First dose	23 (76.7)	No. of adverse events	n = 109
- First and Second dose	7 (23.3)	- Infection / sepsis	21 (19.3)
Number of IRR events	n = 37	- Peripheral sensory neuropathy	11 (10.1)
- Any grade	25 (67.6)	- Pyrexia	8 (7.3)
- Grade 3 or 4	3 (8.1)	- Dyspnoea	7 (6.4)
- Unknown grade	12 (32.4)	- Pneumonia	6 (5.5)
Type of IRR	n = 37	- Cough	6 (5.5)
- Pyrexia	7 (18.9)	- Diarrhoea	5 (4.6)
- Chills/rigors	7 (18.9)	- Constipation	5 (4.6)
- Dyspnoea	5 (13.5)	- Upper respiratory tract infection	5 (4.6)
- Hypotension	5 (13.5)	- Asthenia / fatigue	5 (4.6)
- Pruritus	3 (8.1)	- Insomnia	4 (3.7)
- Vomiting	2 (5.4)	- Nausea / vomiting	3 (2.8)
- Wheezing	2 (5.4)	- Back pain	3 (2.8)
- Others	4 (10.8)	- Delirium	2 (1.8)
Discontinuation due to IRR – n (%)	1 (2.7)	- Others	18 (16.5)
**Hematological adverse events (AEs)**	**N (%)**	- Any grade	17 (15.6)
No hematological AEs	93 (83)	- Grade 3 or 4	7 (6.4)
Hematological AEs	16 (14.3)	- Unknown grade	92 (84.4)
- Thrombocytopenia	7 (43.8)	Permanent discontinuation due to non-hematological AEs	1 (0.9)
- Anemia	6 (37.5)		
- Neutropenia	5 (31.3)		
- Any grade	7 (43.8)		
- Grade 3 or 4	3 (18.8)		
- Unknown grade	9 (56.3)		
Unknown	3 (2.7)		
Permanent discontinuation due to hematological AE	0 (0)		

### e) Stem Cell Harvest Post-Daratumumab Treatment

Nineteen of the 112 patients (17%) comprising 18 with a diagnosis of multiple myeloma and 1 with plasma cell leukemia underwent stem cell harvesting after daratumumab-based therapy. Seventeen (89.5%) achieved adequate stem cell harvests, with yields ranging from 2.5 to 9.18 x10^6^ CD34^+^ cells/kg (median 5.54 x10^6^ CD34^+^ cells/kg). Two myeloma patients, who received concurrent lenalidomide (D-Rd and D-VRd), required plerixafor for mobilisation with stem cell yields of 2.5 and 5.19 x10^6^ CD34^+^ cells/kg, respectively.

## Discussion

Our study demonstrates the tolerability and safety profile of daratumumab among an Asian patient cohort, including a significant proportion (26.7%) with severe renal failure, a group typically excluded from many clinical trials involving daratumumab. We found no significant difference in the ORR (86% versus 76.3%, p = 0.082) or depth of response (≥ CR, 35.1% versus 28.9%, p = 0.469) between patients with and without renal dysfunction. With a quarter of newly diagnosed myeloma patients included, the cohort’s median PL of therapy was only 1, suggesting an inclination toward the early adoption of daratumumab by Singaporean clinicians. Our cohort included a significant proportion (46.5%) of high-risk myeloma patients, with 40.4% exhibiting double or triple-hit disease. This high proportion is partly due to the inclusion of chromosome 1 abnormalities in our definition of high-risk disease,^47^ and partly reflects clinicians’ preference for using daratumumab-based therapies for high-risk patients. Therefore, it would be prudent to exercise caution when comparing studies that do not include this FISH abnormality in their high-risk definitions.

Patients with fewer PL of therapy (0/1 PL versus ≥ 2 PLs) showed better outcomes, with longer median PFS (19.8 versus 6.2 months, p < 0.001) and higher rates of ≥ CR (38.5% versus 16.7%, p = 0.033). High-risk patients with fewer PL also had better ORR (83.3% versus 47.1%, p = 0.022) and achieved similar ORR to the general cohort (83.3% versus 80.2%, p = 0.905), suggesting that daratumumab might overcome high-risk FISH abnormalities if employed either upfront or at first relapse.

This retrospective study has several limitations, some of which are inherent to real-world retrospective studies. We relied on the completeness and accuracy of electronic patient medical records, and data gaps were inevitable due to system migration. To mitigate these issues, we implemented rigorous cross-checking and verification by clinicians. Interpreting adverse events related to daratumumab is challenging, as patients received different agents in combination with daratumumab. It should be noted that our reported rates of grades 3 or 4 hematological and non-hematological adverse events are likely underestimated due to incomplete grading documentation. The relatively short median follow-up (16.9 months) may obscure differences in PFS between subgroups. For similar reasons, the PFS and OS data may not accurately reflect outcomes, especially for newly diagnosed myeloma patients. Nevertheless, our median follow-up duration is comparable to the initial publications of daratumumab-based clinical trials (7.4 months for CASTOR, and 13.5 months for POLLUX).

We identified an ultra-high-risk subgroup of patients with primary refractory disease, of whom 90% harboured high-risk FISH abnormalities, and 60% of them had double or triple-hit myeloma. Despite 70% of these patients receiving a daratumumab-based triplet regimen, incorporating a PI or IMID, their prognosis was dismal. Personalized approaches, including monoclonal antibody-based quadruplet therapies, anti-BCMA antibody-drug conjugates, CAR T-cell therapies, bispecific antibodies, and quadratic phenotypic optimization platform (QPOP), may benefit these patients.^48,49^ Attrition rates are high among myeloma patients, particularly the elderly and frail,^50^ predominantly due to mortality, and the rate increases with each subsequent line of treatment.^51^ Early initiation of daratumumab-based therapy may improve outcomes, and cost-effective analyses of early versus late daratumumab therapy could provide valuable insights for stakeholders.

### Competing Interests

All authors certify that they have no affiliations with or involvement in any organization or entity with any financial interest or non-financial interest in the subject matter or materials discussed in this manuscript.

### Ethics approval and consent

This study was approved by the National Healthcare Group (NHG) Institutional Review Board ethics committee (approval number: (2020/00814). Informed consent was waived by the ethics committee due to the retrospective nature of the study. All procedures performed were in accordance with the ethical standards laid down in the 1964 Declaration of Helsinki and its later amendments.

### Authors’ contribution

All authors contributed to the study conception. A.C.Y. Tso designed and performed the research study, gathered, and analysed the data and wrote the manuscript. S. Acharyya conducted the data analysis and drafted relevant sections of methods and results. WJ Chng, YT Goh, M Ooi, Y Chen, C Nagarajan, D Tan, S Acharyya, and KH Ong contributed data to the study and critically reviewed the manuscript. All authors commented on previous versions of the manuscript and approved the final manuscript.

### Data Availability Statement

Further data will be available upon request to the corresponding author.

## References

[ref-365920] https://www.cancerresearchuk.org/health-professional/cancer-statistics/statistics-by-cancer-type/myeloma#heading-Zero.

[ref-365921] de Mel S., Chen Y., Gopalakrishnan S., Ooi M., Teo C., Tan D.. (2017). The Singapore Myeloma Study Group Consensus Guidelines for the management of patients with multiple myeloma. Singapore Med J.

[ref-365922] Huang J., Chan S.C., Lok V., Zhang L., Lucero-Prisno D., Xu W.. (2022). The epidemiological landscape of multiple myeloma: a global cancer registry estimate of disease burden, risk factors, and temporal trends. Lancet Haematol.

[ref-365923] Barlogie B., Smith L., Alexanian R. (1984). Effective Treatment of Advanced Multiple Myeloma Refractory to Alkylating Agents. N Engl J Med.

[ref-365924] Brenner H., Gondos A., Pulte D. (2008). Recent major improvement in long-term survival of younger patients with multiple myeloma. Blood.

[ref-365925] Usmani S., Ahmadi T., Ng Y., Lam A., Desai A., Potluri R.. (2016). Analysis of Real-World Data on Overall Survival in Multiple Myeloma Patients With ≥3 Prior Lines of Therapy Including a Proteasome Inhibitor (PI) and an Immunomodulatory Drug (IMiD), or Double Refractory to a PI and an IMiD. Oncologist.

[ref-365926] de Weers M., Tai Y., van der Veer M., Bakker J., Vink T., Jacobs D.. (2011). Daratumumab, a novel therapeutic human CD38 monoclonal antibody, induces killing of multiple myeloma and other hematological tumors. J Immunol.

[ref-365927] Overdijk M., Verploegen S., Bögels M., van Egmond M., van Bueren J., Mutis T.. (2015). Antibody-mediated phagocytosis contributes to the anti-tumor activity of the therapeutic antibody daratumumab in lymphoma and multiple myeloma. mAbs.

[ref-365928] Jansen J., Boross P., Overdijk M., van Bueren J., Parren P., Leusen J.. (2012). Daratumumab, a Human CD38 Antibody Induces Apoptosis of Myeloma Tumor Cells Via Fc Receptor-Mediated Crosslinking. Blood.

[ref-365929] Lonial S., Weiss B., Usmani S., Singhal S., Chari A., Bahlis N.. (2016). Daratumumab monotherapy in patients with treatment-refractory multiple myeloma (SIRIUS): an open-label, randomised, phase 2 trial. The Lancet.

[ref-365930] Palumbo A., Chanan-Khan A., Weisel K., Nooka A., Masszi T., Beksac M.. (2016). Daratumumab, Bortezomib and Dexamethasone for Multiple Myeloma. N Engl J Med.

[ref-365931] Dimopoulos M., Oriol A., Nahi H., San-Miguel J., Bahlis N., Usmani S.. (2016). Daratumumab, Lenalidomide, and Dexamethasone for Multiple Myeloma. N Engl J Med.

[ref-365932] Dimopoulos M., Quach H., Mateos M., Landgren O., Leleu X., Siegel D.. (2020). Carfilzomib, dexamethasone, and daratumumab versus carfilzomib and dexamethasone for patients with relapsed or refractory multiple myeloma (CANDOR): results from a randomised, multicentre, open-label, phase 3 study. The Lancet.

[ref-365933] Dimopoulos M., Terpos E., Boccadoro M., Delimpasi S., Beksac M., Katodritou E.. (2021). Daratumumab plus pomalidomide and dexamethasone versus pomalidomide and dexamethasone alone in previously treated multiple myeloma (APOLLO): an open-label, randomised, phase 3 trial. The Lancet Oncology.

[ref-365934] Chari A., Rodriguez-Otero P., McCarthy H., Suzuki K., Hungria V., Balari A.. (2021). Subcutaneous daratumumab plus standard treatment regimens in patients with multiple myeloma across lines of therapy (PLEIADES): an open-label Phase II study. BJH.

[ref-365935] Moreau P., Attal M., Hulin C., Arnulf B., Belhadj K., Benboubker L.. (2019). Bortezomib, thalidomide, and dexamethasone with or without daratumumab before and after autologous stem-cell transplantation for newly diagnosed multiple myeloma (CASSIOPEIA): a randomised, open-label, phase 3 study. Lancet.

[ref-365936] Facon T., Kumar S., Plesner T., Orlowski R., Moreau P., Bahlis N.. (2019). Daratumumab plus Lenalidomide and Dexamethasone for Untreated Myeloma. N Engl J Med.

[ref-365937] Mateos M., Cavo M., Blade J., Dimopoulos M., Suzuki K., Jakubowiak A.. (2020). Overall survival with daratumumab, bortezomib, melphalan, and prednisone in newly diagnosed multiple myeloma (ALCYONE): a randomised, open-label, phase 3 trial. The Lancet.

[ref-365938] Zweegman S., Usman S., Chastain K., Carey J., Ren K., Smith E.. (2019). Bortezomib, lenalidomide, and dexamethasone (VRd) ± daratumumab (DARA) in patients (pts) with newly diagnosed multiple myeloma (NDMM) for whom transplant is not planned as initial therapy: A multicenter, randomized, phase III study (CEPHEUS). JCO.

[ref-365939] Sonneveld P., Dimopoulos M., Boccadoro M., Quach H., Ho P., Beksac M.. (2024). Daratumumab, Bortezomib, Lenalidomide, and Dexamethasone for Multiple Myeloma. N Engl J Med.

[ref-365940] Goldschmidt H., Mai E., Bertsch U., Fenk R., Nievergall E., Tichy D.. (2022). Addition of isatuximab to lenalidomide, bortezomib, and dexamethasone as induction therapy for newly diagnosed, transplantation-eligible patients with multiple myeloma (GMMG-HD7): part 1 of an open-label, multicentre, randomised, active-controlled, phase 3 trial. Lancet Haematol.

[ref-365941] Mateos M., Cavo M., Blade J., Dimopoulos M., Suzuki K., Jakubowiak A.. (2020). Overall survival with daratumumab, bortezomib, melphalan, and prednisone in newly diagnosed multiple myeloma (ALCYONE): a randomised, open-label, phase 3 trial. Lancet.

[ref-365942] Weisel K., Kumar S., Moreau P., Bahlis N., Facon T., Plesner T.. (2023). Daratumumab plus Lenalidomide and dexamethasone (D-RD) versus Lenalidomide and dexamethasone (RD) alone in transplant-ineligible patients with newly diagnosed multiple myeloma (NDMM): Updated analysis of the phase 3 MAIA study. Hemasphere.

[ref-365943] Dimopoulos M., Oriol A., Nahi H., San-Miguel J., Bahlis N., Usmani S.. (2023). Overall Survival With Daratumumab, Lenalidomide, and Dexamethasone in Previously Treated Multiple Myeloma (POLLUX): A Randomized, Open-Label, Phase III Trial. J Clin Oncol.

[ref-365944] Lim S., Spencer A. Putting the best foot forward when treating newly diagnosed multiple myeloma. Internal Medicine Journal.

[ref-365945] Moreau P., Hebraud B., Facon T., Leleu X., Hulin C., Hashim M.. (2021). Front-line daratumumab-VTd versus standard-of care in ASCT-eligible multiple myeloma: matching-adjusted indirect comparison. Immunotherapy.

[ref-365946] Byun J., Park S., Yoon S., Ahn A., Kim M., Lee J.. (2023). Advantage of achieving deep response following frontline daratumumab-VTd compared to VRd in transplant-eligible multiple myeloma: multicenter study. Blood Res.

[ref-365947] Gay F. Results of the Phase III Randomized Iskia Trial: Isatuximab-Carfilzomib-Lenalidomide-Dexamethasone Vs Carfilzomib-Lenalidomide-Dexamethasone As Pre-Transplant Induction and Post-.

[ref-365948] Costa L., Chhabra S., Medvedova E., Dholaria B., Schmidt T., Godby K.. (2022). Daratumumab, Carfilzomib, Lenalidomide, and Dexamethasone With Minimal Residual Disease Response-Adapted Therapy in Newly Diagnosed Multiple Myeloma. J Clin Oncol.

[ref-365949] Munshi N., Avet-Loiseau H., Anderson K., Neri P., Paiva B., Samur M.. (2020). A large meta-analysis establishes the role of MRD negativity in long-term survival outcomes in patients with multiple myeloma. Blood Adv.

[ref-365950] Avet-Loiseau H., Ludwig H., Landgren O., Paiva B., Morris C., Yang H.. (2020). Minimal Residual Disease Status as a Surrogate Endpoint for Progression-Free Survival in Newly Diagnosed Multiple Myeloma Studies: A Meta-analysis. Clin Lymphoma Myeloma Leuk.

[ref-365951] Cavo M., San-Miguel J., Usmani S., Weisel K., Dimopoulos M., Avet-Loiseau H.. (2022). Prognostic value of minimal residual disease negativity in myeloma: combined analysis of POLLUX, CASTOR, ALCYONE, and MAIA. Blood.

[ref-365952] Kastritis E., Palladini G., Minnema M., Wechalekar A., Jaccard A., Lee H.. (2021). Daratumumab-Based Treatment for Immunoglobulin Light-Chain Amyloidosis. N Engl J Med.

[ref-365953] Kastritis E., Theodorakakou F., Roussou M., Psimenou E., Gakiopoulou C., Marinaki S.. (2021). Daratumumab-based therapy for patients with monoclonal gammopathy of renal significance. Br J Haematol.

[ref-365954] Tiew H., Sampath V., Gallardo C., Christopher D., Chan S., Wong S.. (2022). Single-agent daratumumab for refractory POEMS syndrome. AJH.

[ref-365955] Vaxman I., Kumar S., Buadi F., Lacy M., Dingli D., Hayman S.. (2023). Daratumumab, carfilzomib, and pomalidomide for the treatment of POEMS syndrome: The Mayo Clinic Experience. Blood Cancer J.

[ref-365956] Katodritou E., Kastritis E., Dalampira D., Delimpasi S., Spanoudakis E., Labropoulou V.. (2023). Improved survival of patients with primary plasma cell leukemia with VRd or daratumumab-based quadruplets: A multicenter study by the Greek myeloma study group. Am J Hematol.

[ref-365957] Ryu Y., Ricker E., Soderquist C., Francescone M., Lipsky A., Amengual J. (2022). Targeting CD38 with Daratumumab Plus Chemotherapy for Patients with Advanced-Stage Plasmablastoid Large B-Cell Lymphoma. J Clin Med.

[ref-365958] Usmani S., Weiss B., Plesner T., Bahlis N., Belch A., Lonial S.. (2016). Clinical efficacy of daratumumab monotherapy in patients with heavily pretreated relapsed or refractory multiple myeloma. Blood.

[ref-365959] Tso A., Wang S., Gallardo C., Christopher D., Ong K. (2024). Tolerability of Daratumumab Amongst Asian Patients with Plasma Cell Dyscrasias – A Single Centre Experience. Clinical Hematology International.

[ref-365960] Hulin C., Offner F., Moreau P., Roussel M., Belhadj K., Benboubker L.. (2021). Stem cell yield and transplantation in transplant-eligible newly diagnosed multiple myeloma patients receiving daratumumab + bortezomib/thalidomide/dexamethasone in the phase 3 CASSIOPEIA study. Hematologica.

[ref-365961] Lemonakis K., Tatting L., Lisak M., Carlson K., Crafoord J., Blimark C.. (2023). Impact of daratumumab-based induction on stem cell collection parameters in Swedish myeloma patients. Haematologica.

[ref-365962] (2024). https://www.drugs.com/price-guide/mozobil.

[ref-365963] Tan M., Chen Y., Ooi M., de Mel S., Tan D., Soekojo C.. (2023). AL amyloidosis: Singapore Myeloma Study Group consensus guidelines on diagnosis, treatment and management. Ann Acad Med Singap.

[ref-365964] Bayani D., Lin Y., Ooi M., Tso A., Wee H. (2023). Real-world utilization and healthcare costs for multiple myeloma: A retrospective analysis of patients in Singapore. EJHaem.

[ref-365965] (2023). MOH will act to manage cancer drug costs where necessary.

[ref-365966] Kastritis E., Migkou M., Dalampira D., Gavriatopoulou M., Fotiou D., Roussou M.. (2022). Chromosome 1q21 aberrations identify ultra high-risk myeloma with prognostic and clinical implications. Am J Hematol.

[ref-365967] Caro J., Al Hadidi S., Usmani S., Yee A., Raje N., Davies F. (2021). How to Treat High-Risk Myeloma at Diagnosis and Relapse. ASCO education book.

[ref-365968] Rashid M., Toh T., Hooi L., Silva A., Zhang Y., Tan P.. (2018). Optimizing drug combinations against multiple myeloma using a quadratic phenotypic optimization platform (QPOP). Sci. Transl. Med..

[ref-365969] Fonseca R., Usmani S., Mehra M., Slavcev M., He J., Cote S.. (2020). Frontline treatment patterns and attrition rates by subsequent lines of therapy in patients with newly diagnosed multiple myeloma. BMC Cancer.

[ref-365970] McCurdy A., Mian H., LeBlanc R., Jimenez-Zepeda V., Su J., Masih-Khan E.. (2023). Redefining attrition in multiple myeloma (MM): a Canadian Myeloma Research Group (CMRG) analysis. Blood Cancer J.

